# Trained neural networking framework based skin cancer diagnosis and categorization using grey wolf optimization

**DOI:** 10.1038/s41598-024-59979-4

**Published:** 2024-04-24

**Authors:** Amit Kumar K., Satheesha T.Y., Syed Thouheed Ahmed, Sandeep Kumar Mathivanan, Sangeetha Varadhan, Mohd Asif Shah

**Affiliations:** 1 School of Engineering, CMR University, Bengaluru, India; 2 School of Computer Science and Engineering, REVA University, Bengaluru, India; 3 Department of Electrical Engineering, Indian Institute of Technology Hyderabad, Hyderabad, India; 4 School of Computer Science and Engineering, Galgotias University, Greater Noida, 203201 India; 5 Department of Computer Applications, Dr. MGR Educational and Research Institute, Chennai, 600095 India; 6 Kebri Dehar University, Kebri Dehar, Somali 250, Ethiopia; 7 Division of Research and Development, Lovely Professional University, Phagwara, Punjab 144001 India

**Keywords:** Skin cancer detection, Trained neural networks, Federated learning, Feature categorization, Skin cancer, Cancer, Health care, Medical research

## Abstract

Skin Cancer is caused due to the mutational differences in epidermis hormones and patch appearances. Many studies are focused on the design and development of effective approaches in diagnosis and categorization of skin cancer. The decisions are made on independent training dataset under limited editions and scenarios. In this research, the kaggle based datasets are optimized and categorized into a labeled data array towards indexing using Federated learning (FL). The technique is developed on grey wolf optimization algorithm to assure the dataset attribute dependencies are extracted and dimensional mapping is processed. The threshold value validation of the dimensional mapping datasets is effectively optimized and trained under the neural networking framework further expanded via federated learning standards. The technique has demonstrated 95.82% accuracy under GWO technique and 94.9% on inter-combination of Trained Neural Networking (TNN) framework and Recessive Learning (RL) in accuracy.

## Introduction

Skin cancer is the most common type of cancer recorded in United States with an estimate of 145% increase from the current diagnosis statistics. Skin cancer is caused due to pigment abnormalities in outer-layer of skin or epidermis. The process of skin cancer detection and diagnosis is based on the unusual skin textures and predominant symptoms such as bleeding and crusting in the middle of skin wounds, visibility of telangiectasia (small blood vessels). Typically, the melanoma has common symptoms such as skin color change, itching and pain. These signs are inter-correlated with other abnormalities and hence patient attention is missed by many instances. The process of diagnosis and decision classification of skin cancer is based on the primary detection and hence the abnormality ratio of detecting this cancer is in last or complex stage.

Many researchers and research approaches are proposed worldwide to encounter the delay in detection of skin cancer. The primary approach is from the medical community, via improvising the ability to track and categorize the patients based on previous family history, current medical treatment and much more. These precautionary measures are binding experts to provide a higher positive rate of prediction. The medical community has thereby dependent on technological cum biomedical scientist community for the design and development of technological solutions in classifying and predicting the skin cancer based on inter-connecting attributes and parameters. In this research article, an effort is made to propose a trained neural networking framework based skin cancer categorization. The article builds a structural connectivity from one processing technique to another via sharing a trained datasets for effective communication and optimization. The motivation of the proposed technique is to reorganize and structure the existing techniques of skin cancer detection to produce a collective decision support.

The objective of the proposed research is to provide a reliable supporting approach in customization of interdependency parameters in multiple datasets for the processing framework. Since multiple techniques are involved in the process of skin cancer detection, diagnosis and prediction, it makes the system complicated to validate and thereby compute the decision support. The validated datasets are unstructured as the occurrences are based on multiple independent processing units and algorithms. Hence it requires a dedicated framework for skin cancer diagnosis and categorization. The concept of federated learning (FL) is derived from the terminology of distributed computing and centralized decision making. The process and operating principles of FL systems provide an ease in multiple independent dataset coordination and synchronization across multiple platforms and algorithms.

The research article discusses on the probabilities of attributes and feature based diagnosis using grey-wolf optimization approach. The Trained Neural Networking (TNN) framework assures the attribute-feature mapping and the prediction of skin cancer in near future. The article is organized with an introduction and current literature in Sects. “Introduction” and “Proposed methodology”, followed by proposed methodology and problem statement in “Problem Statement” and “Prerequisite and data processing” with a mathematical proof in section “Dimensionality mapping”. The article is concluded with results and discussion, highlights the experimental setup and research findings with a conclusion and scope for future enhancement.

The skin cancer is validated and studied from technological aspect with respect to the primary image processing techniques. The techniques and improvisations of approaches are as discussed in Ref.^1^ computer aided processing and Ref.^2^ with the image processing techniques. The comparative study on image processing based skin cancer detection is drafted by Ref.^3^. Further a noninvasive approach of skin cancer detection technique is discussed in Ref.^4^. These approaches are treated as the building blocks of technological techniques in resolving global skin cancer diagnosis. The process of diagnosis is improvised with neural networking based frameworks, followed with deep learning and artificial intelligence^5,6^. The deep learning and neural networking techniques has successfully outperformed image processing techniques in performance and accuracy detection.

Device based interference is computed and validated^7^ with a dedicated Convolutional neural network (CNN) and application interference. The bioinspired algorithms are boon in solving the skin cancer diagnosis. Practical swarm optimization (PSO) is designed to improvise the feature selection process from the attribute pool of training dataset^8,9^. The biomedical datasets are considered for longer validations under multiple attributes detainment ratio^10^. The supporting clinical trials and processing of skin cancer based detection is validated by sound analysis algorithms. The process of deep learning based attribute matching and proposed attribute validation is supported for mapping and categorizing the most influential feature in providing an accurate decision support^11^. The validation of supporting the immune system based cancer analysis is reported by Ref.^12^ under osteoporosis.

The proposed framework is designed and validated with optimizing algorithms for performance improvisation^13,14^. These algorithms designed are based on dataset attribute-feature dependency matching. The features are reduced with dimensions to upgrade the selected attributes in providing a reliable decision support. The approach of Convolutional Neural Networking (CNN) provides a reliable classification of skin cancer^15^. The approach discusses various across-domain studies and techniques used in classification. The further evolution of CNN is Deep-CNN (DCNN) models^16^. The process of DCNN is enhanced using an additional customization approach using transfer learning. The transfer learning based systems are customized with inter and cross-domain learning of datasets.^17^ The Bayesian Deep Learning (BDL) models are one such models with Three-way decision validations of skin cancer datasets. The BDL is implemented on uncertainty quantification (UQ) methods. The principle of robustness in the dynamic datasets are at peak of computation and relevance.

The inclusiveness of transfer learning is enhanced by Ref.^18^ in benchmark setting and out-of-distribution (OOD) testing. This includes a wide-range of datasets from multiple sources and archives. The validation of skin cancer classification and decision making is further represented in Ref.^19^ with respect to the performance efficiency and technological categorization. The summary of this survey is to provide an inclusive overview in categorizing and customizing the data-attribute ratios using optimization algorithms for skin cancer classification and diagnosis. In Ref.^20^ the novel approach using sliding window technique is proposed with a recorded accuracy of 97.8% in skin cancer prediction. The technique has further included Concentrated-Xception-ResNet50 models. The studies discuss an improvised approaches such as augmented intelligence^21^ and multilevel threshold segmentation^22^ and a detailed review is reported by Ref.^23^ highlighting the multiple dimensionalities of dataset processing, methodologies, feature extracting techniques and much more.

In recent times a novel and effective optimizations technique such as Liver Cancer Algorithm (LCA)^24^, Harris Hawks optimization (HHO)^25^ and RIME optimization algorithm (RIME)^26^ are proposed and discusses the versatile properties of bio-inspired algorithms computation for optimizing the features. Whereas the in the proposed technique we have considered to use the Grey-Wolf Optimization^27^ based technique for categorizing and customizing the skin cancer features.

## Proposed methodology

**Figure 1:**
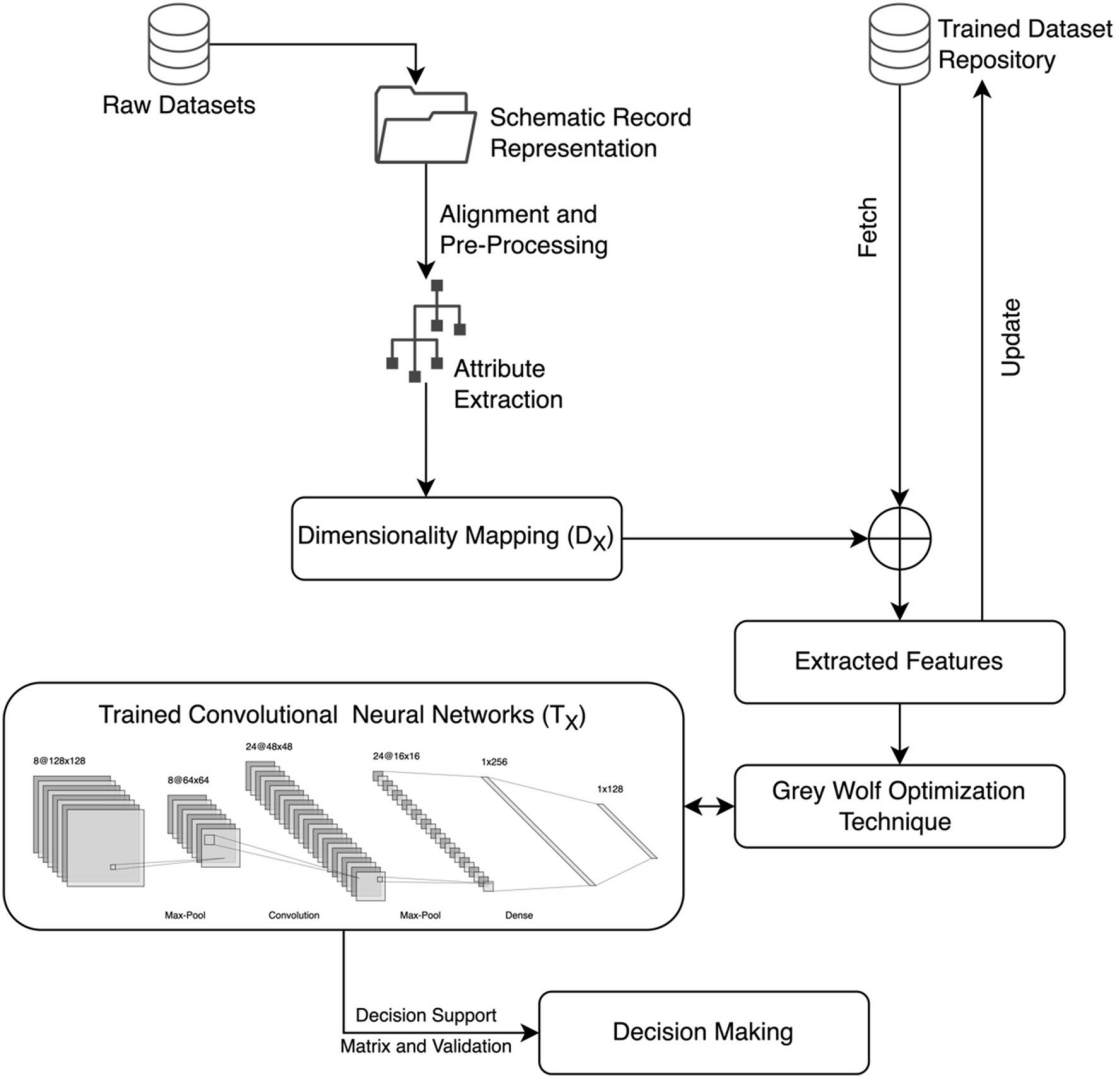
Dataset dimensionality mapping and feature optimization model.

The proposed methodology is designed and developed with the objective to provide a reliable solution for early detection and classification of skin cancer. The technique includes the trained dataset repository from the existing sources and algorithms (ISIC 2020 Sydney Melanoma Diagnosis Centre and Melanoma Institute Australia, HAM and SKINL2 2019. The purpose is to extract threshold value attenuation ratio from processed datasets in providing accurate probability for decision support. The initial pre-processed datasets are represented in schematic records. These records are primary elements for processing data standardization and alignment. The schematic records extract attributes from raw datasets (trained repositories) to provide a dimensional representation matrix of primary attributes. The matrix consists of attributes and feature coordination ratio such as geographical reporting, gender, age, lesion size, area, primary comorbidities and patient history records and hence dimensionality mapping is required to assure attribute to feature correlation is achieved as shown in Fig. 1. The inter association of mapped raw attribute with trained datasets repository provide a stable format of attribute-feature ratios. In Fig. 1, the methodology transforms information parameters to a trained CNN model to attain a decision support in detecting skin cancer. The Table 1 represent a detailed representation of mathematical model for ease in understandability.

**Table 1:** Mathematical terms representation.

Symbol	Description
$\left( {D_{X} } \right)$	Trained repository datasets
$\left( {\Delta {\rm T}} \right)$	Training arbitrary
$\left( \omega \right)$ and $\left\| {\Delta \omega } \right\|$	Weight matrix
$\left( \eta \right)$	Neutralization factor
$\left( \varepsilon \right)$	Elimination matrix
$\left\| {D_{P} } \right\|$	Dataset variable under dimensionality matrix
$\left( L \right)$	Layer dimension
$\left( {{\rm T}_{X} } \right)$	Trained convolutional neural networks
$\left( {O_{G} } \right)$	Grey wolf optimization

**Figure 2:**
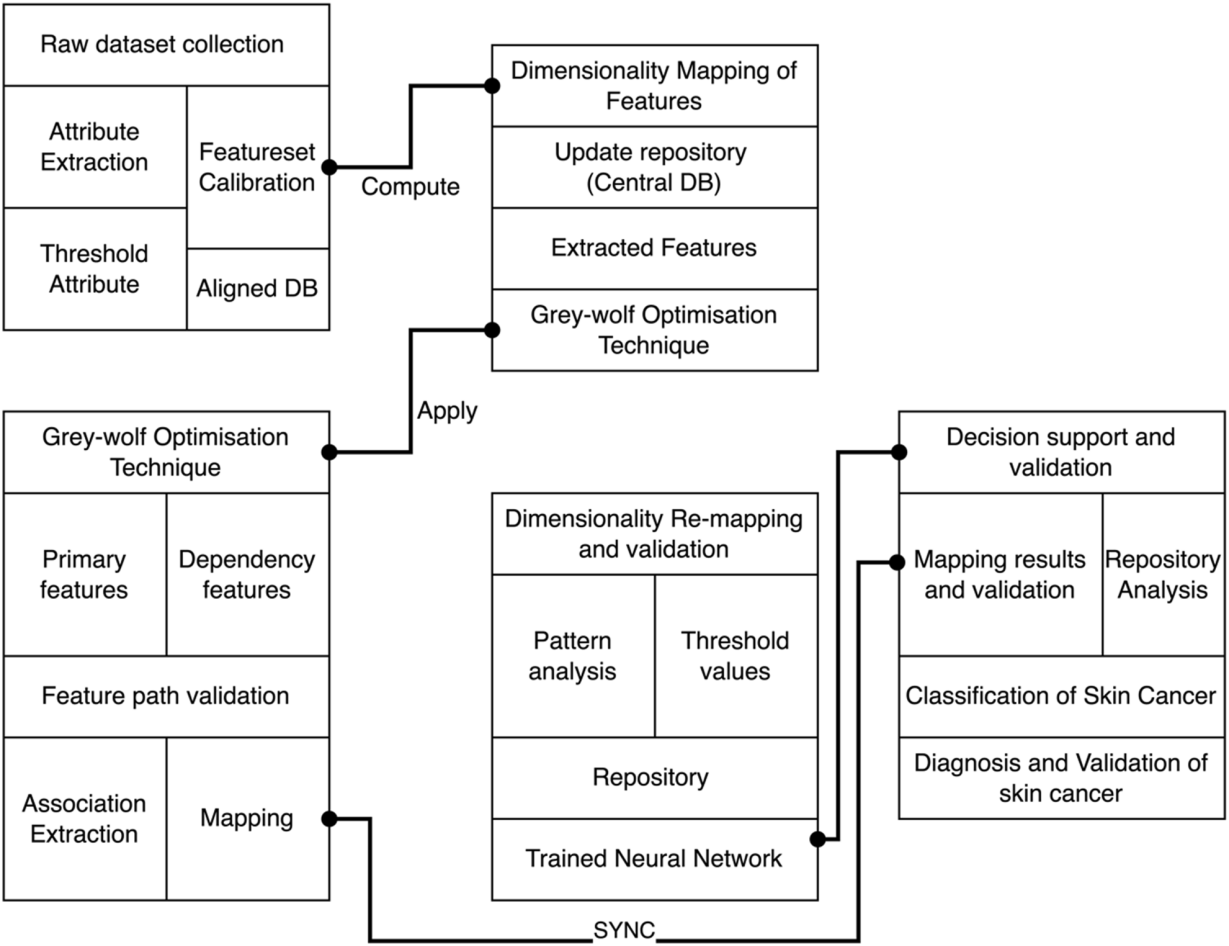
Interdependency representation and classification for decision making.

On successfully extracting the attributes from inter-relationship mapping and feature extraction as shown from Table 2 dataset, Grey Wolf Optimization (GWO) algorithm is processed and appended. The GWO extracts most significant feature and attribute from the extracted attribute-feature pool as demonstrated in Fig. 2. The process is to obtain the most significant and influential feature of skin pattern such as lesion size, rate of spread and area of interest to provide a reliable decision making via Trained Convolutional Neural Networks (TCNN). The TCNN is designed based on feedback layer and update based training. The attribute and feature mapping with an impact on decision support and validation is described in classification diagram (Fig. 2). The process of decision making and validation of skin cancer classification is based on secondary mapping and synchronization. The process of mapping coordination and the repository analysis assures higher performance of decision support.

**Table 2:** Skin cancer trained repository datasets and feature matrix.

Dataset Name	Country	Year of publication	Skin cancer lesions included	Number of participants	Number of images
ISIC 2020 Sydney Melanoma Diagnosis Centre and Melanoma Institute Australia4^23^	Australia	2020	8	441	1884
ISIC 2020 Memorial Sloan Kettering Cancer Centre^23^	USA	2020	5	523	11,108
SKINL2^25^	Portugal	2019	8	Not reported	814
HAM10,000^24^	Austria and Australia	2018	8	Not reported	10,015

The Fig. 2 represents the classification model for interdependency representation of skin cancer computation as discussed in Fig. 1 of the proposed system. The Fig. 2 further demonstrates the purpose of extracting features and correlating with the datasets with respect to the computational developments. The classification is based on phases of the implementation such as raw dataset collection, dimensionality mapping of features, Gray-wolf optimization technique and decision support. Typically, the individual unit represents the operations and tanks undertaken.

## Problem statement

The validation of skin cancer detection and categorization is currently processed by the Convolutional Neural Networking (CNN) framework. Typically, these neural networks are aligned and calibrated with training datasets. The dataset is static and freeze (pre-loaded) before the initialization of the process and are centralized in nature. The process of data centralization is due to the trivial computational approaches of processing. This results in data-indexing and heap-order repository creation causing a larger volume of data deposits. The further results lead to ineffective computation and false-positive attribution on decision making hence, the dataset modification and updates leads the neural network to unlearn the decision support. Consider the dataset $\left( {D_{X} } \right)$ as the trained repository with $\left( {x_{i} } \right)$ attributes lined for computational validation. The approach technically derives $\left( {D_{X} \Rightarrow \infty } \right)$. If and only if the optimized attributes $\left( {x_{j} } \right)$ is extracted as $\left( {x_{j} \subseteq x_{i} } \right)$ and $\left( {x_{j} \Rightarrow D_{X} \Rightarrow \infty } \right)$. Hence the orientation of changing values functions is fluctuated with changing time internals.

The validation of the attributes $\left[ {\left( {V_{x} } \right) \Rightarrow \left( {x_{j} \cup D_{X} } \right)_{0}^{n} } \right]$ such that $\left( {\forall x_{i} x_{j} \in D_{X} } \right)$ with changing dataset under training arbitrary $\left( {\Delta {\rm T}} \right)$ as shown in Eq. (1) with summation function of each independent variable associated and managed with training datasets $\left( {\Delta {\rm T}} \right)$.

1 $$\Delta {\rm T} = \sum\limits_{i,j}^{n} {\left[ {f\left( {x_{i} \left( {x_{j} } \right)} \right) \cup \left( {D_{X} } \right)_{i,j} } \right]} \Rightarrow \infty$$

The major challenge on dataset processing is associated with the phase of training and evaluation. The solution for the approach is to rectify the training dataset with a dedicated neural networking framework operated in the source of data origin.

## Prerequisite and data processing

In the context of skin cancer datasets, data preprocessing plays a vital role in preparing the data for analysis and machine learning applications. Initially, data cleaning techniques are employed to address missing values and outliers, ensuring data integrity. Subsequently, data transformation steps are applied to encode categorical variables, scale numerical features, and handle data normalization. Feature selection and extraction methods are utilized to identify relevant features and reduce dimensionality, enhancing the efficiency of subsequent analyses. Data integration techniques consolidate information from diverse sources, while data reduction methods help manage large datasets. Normalization ensures consistency in feature scales, crucial for accurate model training. Finally, the dataset is split into training, validation, and test sets to facilitate model development, parameter tuning, and evaluation. By systematically preprocessing skin cancer datasets, researchers can optimize data quality and facilitate more robust analysis, ultimately advancing our understanding and management of skin cancer. The skin cancer dataset is aligned and contributed by the National Cancer Imaging (NCI) and Cancer Image Archive (CIA) institute archives. The datasets are also aligned with Kaggle repository for validation using random processing. The CIA datasets are used in 60:40 training and testing validation, whereas the kaggle based cancer_datasets are used for independent validation. The NCI dataset considered are with 1274 maligned and 1173 benign pre-trained and the performance sources are pre-acquired with the training data technique. Consider the multisource repository alignment of datasets as $\left( {D_{X} } \right)$ with each $\left( {D_{X} = D_{1} ,D_{2} ,D_{3} .......D_{X} } \right)$ the xth value of $\left( {\Delta D_{X} } \right)$ is reflected and attributed with the associated attributes $\left( {A_{i} } \right)$ as shown in Eq. (2) with weights $\left( \omega \right)$

2 $$\sum D_{i} = \varepsilon \left[ {\eta \sum\limits_{i = 1}^{n} {\sum\limits_{j = i + 1}^{n} {\omega \left( {A_{i} \left[ {D_{j} } \right]} \right)} } } \right]$$

3 $$\therefore \sum D_{i} = \eta \sum\limits_{i = 1}^{n} {\sum\limits_{j = i + 1}^{n} {\omega_{i} \left\{ {\varepsilon \left( {A_{i} \left[ {D_{j} } \right]} \right)} \right\}} }$$

where $\left( \eta \right)$ is the neutralization factor for collective processing with $\left( \varepsilon \right)$ as elimination matrix as shown in Eqs. (2) and (3) respectively. The acquired image segments are further retained and processed with weight calibration and fragmentation. The resultant weight matrix $\left( {\omega \left( {n \times m} \right)_{i} } \right)$ is a relevance to assure the data streams are interconnected and attribute mapping network is optimized.

## Dimensionality mapping

The acquired dataset $\left( {D_{X} } \right)$ with an optimized attribute association $\left( {\Delta D_{i} } \right)$ is represented as shown in Eq. (3). The relevance of weight $\left( \omega \right)$ association is framed under $\varepsilon \left( \omega \right)$ with an interdependency mapping as $\left( {\varepsilon \left| \omega \right| = \left\| {\Delta D_{i} } \right\|} \right)$ at the given data intervals. The technical differences of the optimized data and relevance parameters are mapped towards dimension reduction. The dimensional paradigms are further influential in capturing attributes $\left( {\Delta D_{i} } \right)$ to dimensional type mapping as shown in Eq. (4)

4 $$\varepsilon \left\| {D_{P} } \right\| = \sum\limits_{i}^{\infty } {\prod\limits_{j = i + 1}^{n} {\left[ {\omega_{{\left( {i,j} \right)}} \Rightarrow \frac{{\delta \left( {\varepsilon \left| \omega \right|} \right)_{i} }}{\delta t} \times \frac{\delta \left( t \right)}{{\delta \left( {\Delta D_{j} } \right)}}} \right]} }$$

5 $$\therefore \varepsilon \left\| {D_{P} } \right\| = \sum\limits_{i}^{\infty } {\prod\limits_{j = i + 1}^{n} {\left[ {\omega_{{\left( {i,j} \right)}} \Rightarrow \frac{{\delta \left( {\varepsilon \left| \omega \right|} \right)_{i} }}{{\delta \left( {\Delta D_{j} } \right)}} \times \Delta t} \right]} }$$

where, the functional representation of data variables under dimensions $\left\| {D_{P} } \right\|$ is evaluated with an elementary matrix $\left( \varepsilon \right)$. The process is aligned with weight matrix and a data type prediction ratio as shown in Eq. (4). The variation matrix of skin cancer dataset $\left( {\Delta D_{j} } \right)_{0}^{S}$ is further aligned to the optimized matrix in Eq. (5) with extensive representation as shown in Eq. 6.

6 $$\therefore \varepsilon \left\| {D_{P} } \right\|_{0}^{S} = \sum\limits_{i}^{{}} {\prod\limits_{j \to i}^{n} {\left[ {\left\| {\omega_{{\left( {i,j} \right)}} } \right\| \Rightarrow \frac{{\delta \left( {\varepsilon \left| \omega \right|} \right)_{i} \cup \delta \left( {D_{j} } \right)_{0}^{S} }}{{\delta \left( {\Delta D_{j} } \right)}} \times \Delta {\rm T}} \right]} }$$

On retaining the time matrix with reference to Eq. (6), the weight matrix $\left\| {\omega_{{\left( {i,j} \right)}} } \right\|$ is dependent for computation in relatively aligned manner. The elementary matrix is reduced and limited to the operations of $\left( {\varepsilon \left\| {D_{P} } \right\|_{0}^{S} } \right)$ with $\left( {0 \to S} \right)$ iteration. The further dimension reduction is optimized and represented as in Eq. (7).

7 $$\sum \left\| {D_{P} } \right\|_{0}^{S} \Rightarrow \prod\limits_{{\left( {i,j} \right) \to S}}^{n} {\left[ {\frac{{\delta \left( {\varepsilon \left| \omega \right|} \right)_{i} \cup \delta \left( {\Delta D_{j} } \right)_{0}^{S} }}{\delta t}} \right]} \times \frac{{\Delta {\rm T}}}{L}$$

where $\left( L \right)$ is the layer dimension processing under defined matrix representation of $\left\| {D_{P} } \right\|$. The overall representation of dimension is collectively focused in the optimization of processing datasets.

## Trained convolutional neural networking framework for dimensionality mapping

On extraction of dimensional optimization matrix in the Eq. (7), the further processing is reflected with respect to mapping. The process of dimensionality mapping $\left( {D\left[ M \right]_{0}^{S} } \right)$ is representative function of multiple values and framesets with RoI based extraction $\left( {{\mathbb{R}}_{Z} } \right)$. The layering $\left( L \right)$ with summarization matrix $\left( {S_{M} } \right)$ is represented as shown in Eq. (8)

8 $$L = \prod\limits_{{\left( {i,j} \right) \to S}}^{n} {\left[ {\sum\limits_{k} {\left( {\left\| {\omega_{{\left( {i,j} \right)}} } \right\|\eta \left( {L_{i} } \right)_{k} - \sum {\left( {\frac{{\left\| {S_{m} } \right\|_{k} }}{{{\mathbb{R}}_{Z} }}} \right)} } \right)} } \right]}$$

9 $$\therefore \sum \left( L \right) = \prod\limits_{{\left( {i,j} \right) \to S}}^{n} {\left[ {\frac{{\partial \left( {{\mathbb{R}}_{Z} } \right)\Delta {\rm T}}}{0.7023}\left( {\left\| {\omega_{{\left( {i,j} \right)}} } \right\|\eta \left( {L_{i} } \right)_{k} - \sum {\left( {\frac{{\left\| {S_{m} } \right\|_{k} }}{{{\mathbb{R}}_{Z} }}} \right)} } \right)} \right]}$$

10 $$\therefore \sum \left( L \right)_{k} = \prod\limits_{{\left( {i,j} \right) \to S}}^{n} {\left[ {\frac{{\partial \left( {{\mathbb{R}}_{Z} } \right)\Delta {\rm T}}}{0.7023}\left( {\left\| {\omega_{{\left( {i,j} \right)}} } \right\|\eta \left( {L_{i} } \right)_{k} - \sum\limits_{k \to S}^{n} {\left( {\left\| {S_{m} } \right\|_{k} } \right)} } \right)} \right]}$$

On Eq. (9), the possibility of functionality vector $\left( L \right)$ and the RoI $\left( {\mathbb{R}} \right)$ range is computed. The factorial representation is layered and the functional aspect of $\sum \left( L \right)$ is computed with reference to Eq. (10). Typically, the processing vector component is aligned using $\left( {\left\| {S_{m} } \right\|_{k} } \right)$ to an interconnected ratio attribute. The extracted cum optimized layer $\left[ {\sum \left( L \right)_{k} } \right]$ is reflective in the attribute $\left( A \right)$ and hence the mapping $\left. {D\left( m \right)} \right)_{0}^{s}$ is represented in Eq. (11)

11 $$\left. {D\left( m \right)} \right]_{0}^{S} = \eta W_{{\left( {i,j} \right)}} - \sum\limits_{i}^{n} {\sum\limits_{j}^{{}} {\left( {\frac{{\delta \left[ {L_{k} } \right]_{i} \cup \delta \left[ {D_{P} } \right]_{j} }}{\delta t}} \right)} }$$

12 $$\left. {\therefore D\left( m \right)} \right]_{0}^{S} = \eta \frac{{\left\| {\Delta \omega } \right\|}}{{\Delta {\rm T}}} - \sum\limits_{i}^{n} {\sum\limits_{j}^{{}} {\left( {\frac{{\delta \left[ {L_{k} } \right]_{i} \cup \delta \left[ {D_{P} } \right]_{j} }}{\delta t}} \right)} }$$

According to Eq. (11), the coordination of weight matrix $\left\| {\Delta \omega } \right\|$ is reflected with the association of $\left\| {\Delta {\rm T}} \right\|$ values for suturing the dataset recommendation. The process is further computed with the layer $\left[ {\sum \left( L \right)_{k} } \right]$ and $\left. {D_{P} } \right)_{0}^{s}$ at the instance of operation. The attribute of skin cancer layering and $\left( {{\mathbb{R}}_{Z} } \right)$ RoI extraction is based on dependency. Technically, the formulation of multiple layers and associations are streamlined in mapping function as represented in Eq. (12).

The mapping order of skin cancer dataset $\left( {D_{X} } \right)$ under $\left( {\Delta D_{f} } \right)$ is optimized and mapped for representational purpose. The outcome of rational mapping is resultant of trained neural network $\left( {{\rm T}_{X} } \right)$ as represented in Eq. (13).

The terminology of trained neural network (TNN) is based on feedback competitive learning models. The approach segments the dataset $\left( {D_{X} } \right)$ into multiple layers as input layer, hidden layer, computational layer and feedback layer. Typically, the process includes a structural representation of feedback based self-learning models. The overall streamlining $\sum \left( {\left. {D\left( m \right)} \right]_{0}^{S} } \right)$ is evaluated within the scope of mapping range as shown in Eq. (13).

13 $${\rm T}_{X} = \left( {\sum A + f\left( {D_{S} } \right)_{i} } \right) + \left( {\sum B + f\left( {D_{S} } \right)_{i + 1} } \right) + ..........$$

14 $$\therefore {\rm T}_{X} = \left[ {A + \sum\limits_{i}^{n} {f\left( {D_{S} } \right)_{i} } } \right] \cup \left[ {D\left( {\rm M} \right)_{0}^{S} } \right]$$

The relevance ratio of training neural networking based $\left( {{\rm T}_{X} } \right)$ is optimized and summarized with reference to $f\left( {D_{S} } \right)$ as the feature set and the occurrence pattern. The representation of $\left[ {D\left( {\rm M} \right)_{0}^{S} } \right]$ is associated to the i^th^ value of overall nodes (n) involved in TNN processing. The outcome of differences is subjected $\sum \left[ {D\left( {\rm M} \right)_{0}^{S} } \right]$ with a decision support for categorization.

## Grey-wolf optimization approach

The extracted layers and dimensions are classified and processed with a decision support. The decision is subjected with attribute and dataset optimization. The TNN framework assures the reliability of feedback based self-learning environment. The grey-wolf optimization $\left( {O_{G} } \right)$ is to reduce and provide the ranging attributes associated with $\left( {{\rm T}_{X} } \right)$ and $\left[ {\left. {D_{{\left( {\rm P} \right)}} } \right|_{0}^{S} } \right]$ such that, $\left[ {\forall \left\| {D_{\rm P} } \right\|_{0}^{S} \Rightarrow \sum {\rm T}_{X} } \right]$ and $\left( {{\rm T}_{X} \notin D_{X} } \right)$ at initial and final computational stages. The process of alignment is subjected to the variation of parameters in attribute $\left( {A_{i} } \right)$ such that $\left( {\forall A_{i} \Rightarrow D_{X} } \right)$ and $\left( {A_{i} \subseteq D_{X} } \right)$ at an outset, the formulation vector is computed as shown in Eq. (15).

15 $$O_{G} = \mathop {\lim }\limits_{\Delta S \to \infty } \left[ {\frac{{\delta \left( {\Delta {\rm T}_{X} } \right)}}{\delta t} \otimes \omega \left( {\left\| {D_{\rm P} } \right\|_{0}^{S} } \right)} \right]$$

16 $$\therefore O_{G} = \mathop {\lim }\limits_{\Delta S \to \infty } \left[ {\prod\limits_{i}^{\eta } {\sum\limits_{j}^{S} {\left( {\frac{{\delta \left( {\Delta {\rm T}_{X} } \right)_{{\left( {i,j} \right)}} }}{\delta t}} \right)} } \otimes \omega \left( {\left\| {D_{\rm P} } \right\|_{0}^{S} } \right)} \right]$$

The grey wolf optimization $\left( {O_{G} } \right)$ is appended on the trained neural networking framework for collective processing. The weight of association is aligned with $\left( {\left\| {D_{\rm P} } \right\|_{0}^{S} } \right)$ such that, $\left( {\forall \left\| {D_{\rm P} } \right\|_{0}^{S} \Rightarrow \Delta {\rm T}\left( {O_{G} } \right)_{Z} } \right)$, where $\left( x \right)$ is the functional variable of optimized techniques.

17 $${\mathbb{R}}_{Z} = \mathop {\lim }\limits_{n \to \infty } \left( {\frac{{L\left( {\left\| {D_{\rm P} } \right\|_{0}^{S} } \right)}}{L\left( t \right)} \cup \mathop {\lim }\limits_{n \to S} \left( {\frac{{\partial^{2} \left( {O_{G} } \right)_{x} }}{{\partial t^{2} }}} \right) \oplus \Delta {\rm T}} \right)$$

18 $$\therefore {\mathbb{R}}_{Z} = \mathop {\lim }\limits_{n \to \infty } \left( {\frac{{L\left( {\left\| {D_{\rm P} } \right\|_{0}^{S} } \right)}}{L\left( t \right)} \cup \prod\limits_{j = 1}^{\eta } {\sum\limits_{k = j + 1}^{S} {\left( {\frac{{\partial^{2} \left( {O_{G} } \right)_{j} \cap \partial^{2} \left( {D_{X} } \right)_{k} }}{{\partial t^{2} }}} \right)} } \oplus \Delta {\rm T}} \right)$$

According to Eqs. (17) and (18) the formulation of datasets $\left( {\left\| {D_{\rm P} } \right\|_{0}^{S} } \right)$ is bound with respect to the optimization $\left( {O_{G} } \right)$ under a constant recurrence format. Typically, the functional representation of $\left( {\left\| {D_{\rm P} } \right\|_{0}^{S} } \right)$ under a recurrence ration is evaluated for effective computations. The order of evaluation and occurrence ratio of dataset $\left( {D_{X} } \right)$ is further subjected with the inter-common attribute and feature elimination as shown in Eq. (18). The buffer factor of $\left( {\Delta {\rm T}} \right)$ is associated to assure the saturation of threshold values in the frame of dataset.

## Results and discussions

**Figure 3:**
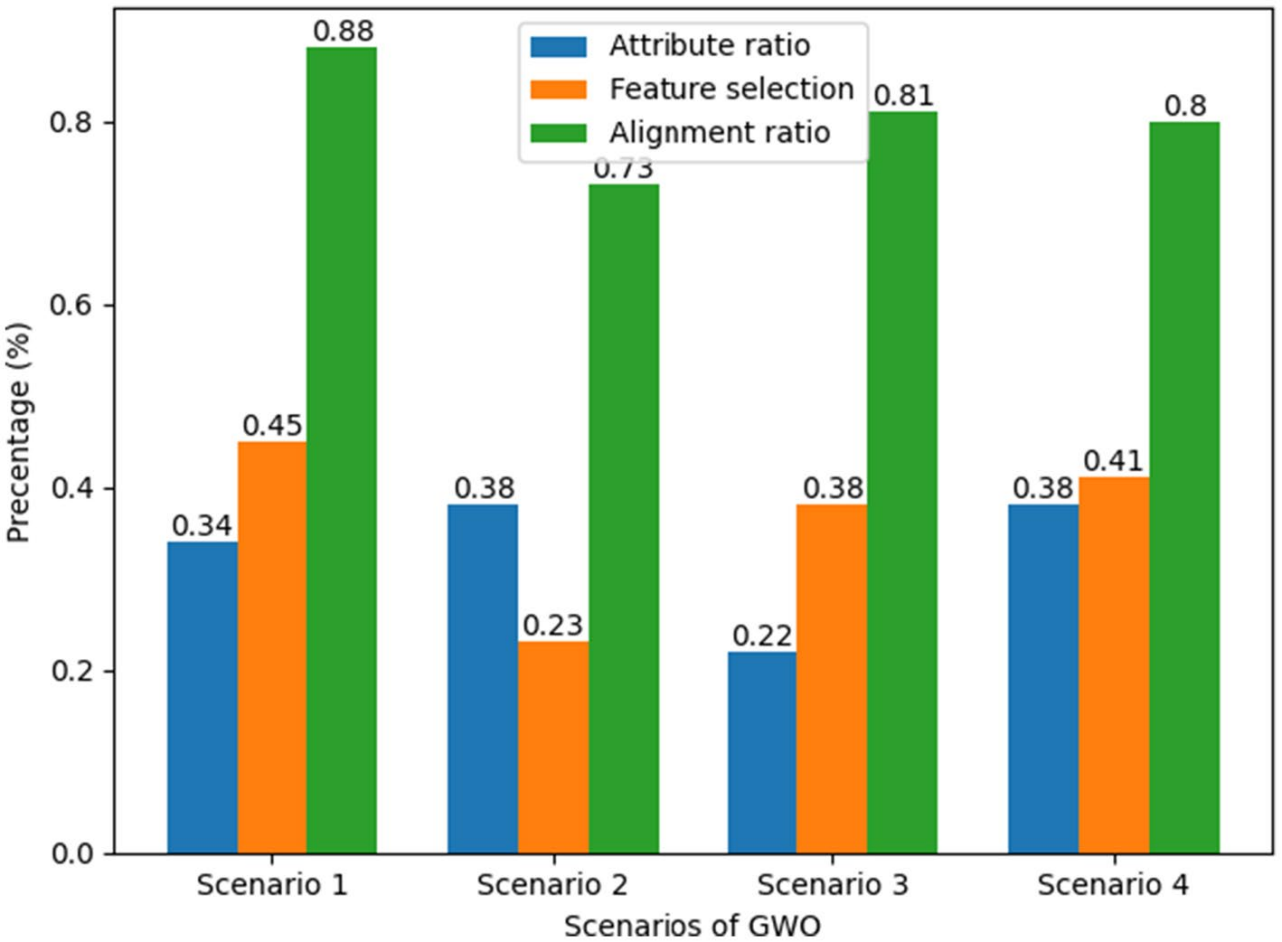
Attribute ratio extraction and evaluation parameter comparison.

The proposed technique of skin cancer classification and validation is supported on the dynamic datasets of pre-processed and trained datasets. The process has included raw dataset schematic record based feature extraction and mapping with reference to trained dataset repository. The process of attribute ratio extraction on multiple scenarios are demonstrated in Fig. 3. The alignment rations of each independent attributes are correlated and functioned into a feature matrix to provide an alignment ratio of interconnected values. This includes the performance efficiency of attribute ratio with respect to feature selection for the give interval of values with reference to alignment ratio. The scenario is based on the split ratio of training and testing datasets.

**Figure 4:**
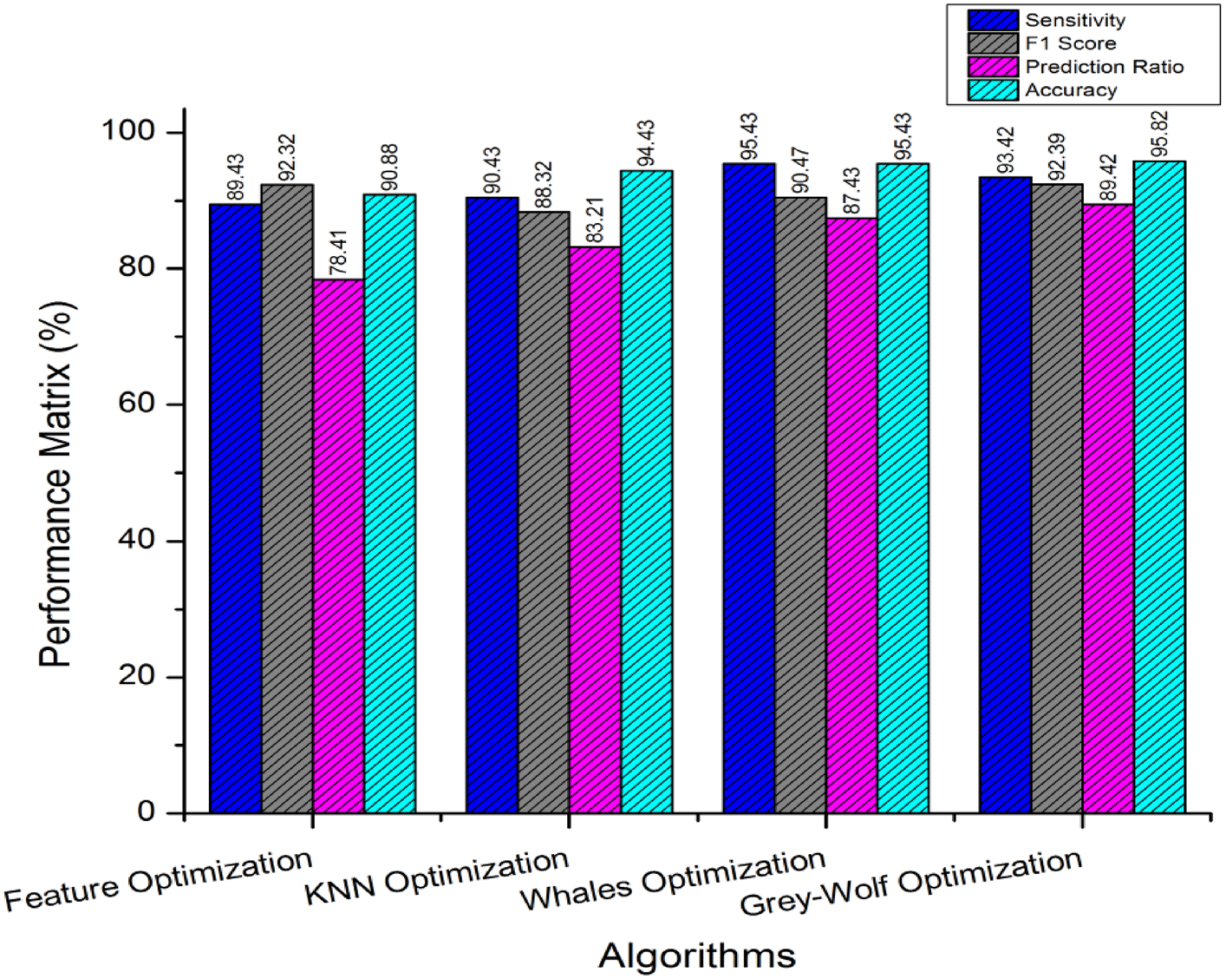
Comparison with optimization algorithms.

Figure 4 discusses the performance outcomes of proposed Grey-wolf Optimization (GWO) technique over the existing approaches such as feature optimization, KNN optimization and whale’s optimization. The outcome of proposed GWO based feature optimization has improved and outperformed to 95.82% in accuracy compared to the other techniques.

**Figure 5:**
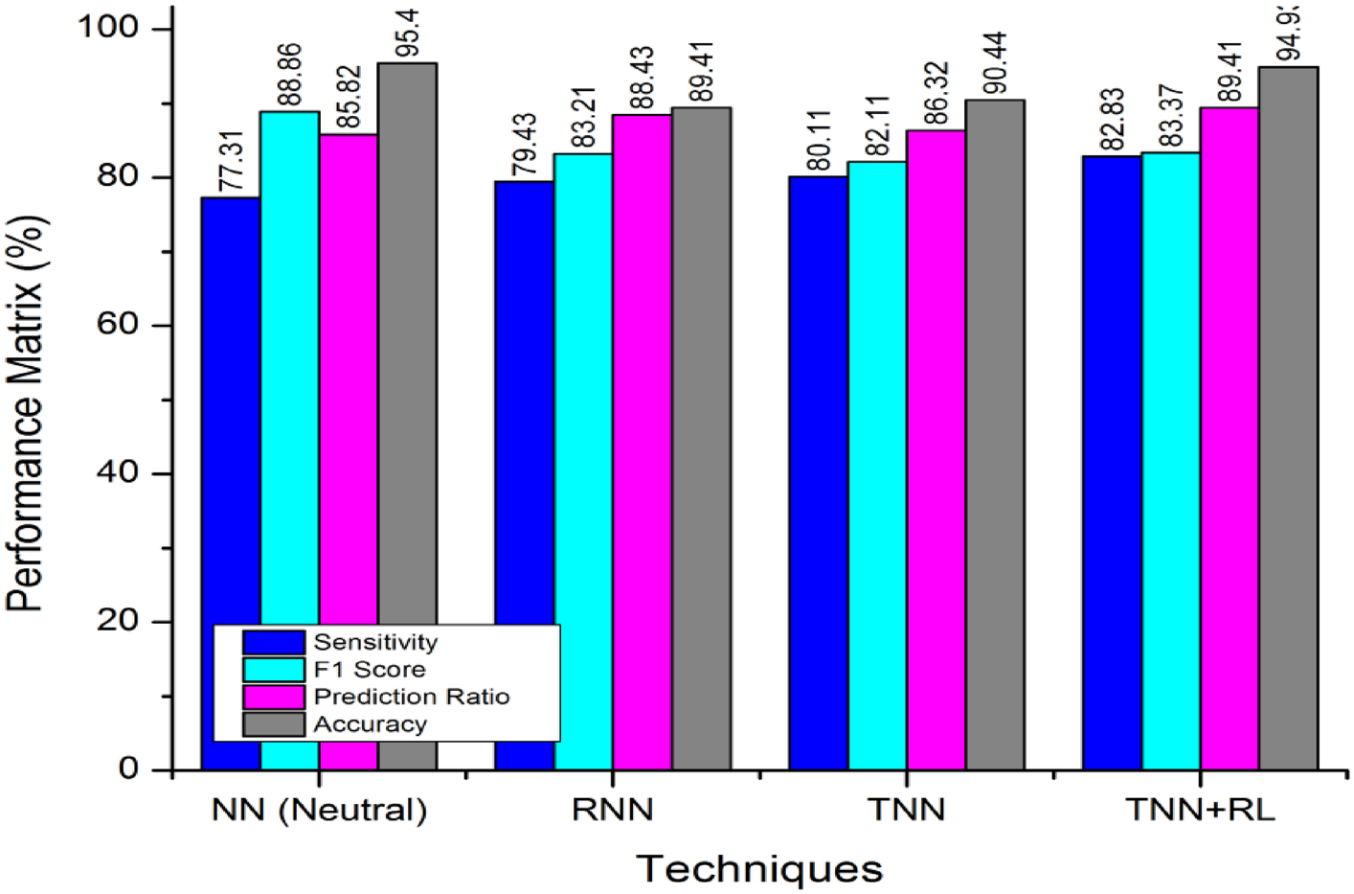
Observation and study evaluation with reference to trained neural networking technique.

According to the extracted values of multiple comparison as shown in Fig. 5, the proposed Trained Neural Networking (TNN) framework is evaluated under the Recursive Learning (RL). The TNN + RL based computation has increased the probability of decision making and supporting. The RL adds-on the feedback layer with structural updates of processing (hidden layers) from the TNN model. The ratio of True-Negative (TN) over False Positive (FP) is bounded with a minimal navies approach for optimizing the prediction and classification ratio.

The anticipated outcomes undergo comparison and validation using various cross-domain methodologies such as K-nearest neighbors (KNN), whale optimization, and Grey-wolf optimization. This process entails independent dataset processing and decision-making capabilities. The performance varies across instances, transitioning from KNN optimization to whale optimization. These optimization techniques are inherently system-driven, with datasets being preserved and centralized. In contrast, the proposed Grey-wolf optimization is tailored to Tensor-based neural network (TNN) recursive learning models within federated systems. The federated learning framework operates primarily in a decentralized manner and exhibits a higher computational ratio compared to existing approaches. The Grey-wolf optimization mechanism is refined through inter-domain computation utilizing federated learning (FL) and reinforcement learning (RL) models of TNN.

The approach outlined presents several potential limitations that merit consideration. Firstly, the reliance on specific optimization techniques like KNN, whale optimization, and Grey-wolf optimization may restrict the generalizability of results, as these methods might not universally suit all datasets or problem domains. Moreover, while KNN and whale optimization are depicted as centralized methods with centralized datasets, this centralized nature could be problematic for applications involving distributed or privacy-sensitive data. Additionally, the complexity of Grey-wolf optimization, particularly when applied to TNN-based recursive learning models in federated systems, may introduce implementation challenges and hinder its effectiveness. The computational demands of the federated learning framework, compounded by the inclusion of Grey-wolf optimization, could pose significant computational burdens. Furthermore, the efficiency and effectiveness of inter-domain computing leveraging FL and RL models of TNN to enhance Grey-wolf optimization may be limited by factors such as data heterogeneity or communication overhead. Lastly, the generalizability of performance comparisons across different optimization techniques may not extend to all datasets or real-world scenarios, necessitating careful consideration of dataset characteristics and scalability concerns.

## Conclusion

The proposed technique is evaluated on the process of diagnosis and classification of skin cancer datasets into a categorization. The technique has achieved the process of evaluation and feature classification based on trained neural networking framework. The extracted and trained neural network has outperformed the existing techniques. The dual process of validation is aligned and processed with grey-wolf optimization technique in further categorization of RoI and provide a reliable decision support. The technique has retrieved and classified datasets under 60% training and 40% testing module. Overall the proposed technique has extracted an accuracy of 94.9% with the skin cancer images and classification based on pattern extracted. In near future, the technique can be validated on dynamic Artificial Neural Networks (dANN).

## Data Availability

The datasets used during the current study are available from the corresponding author on reasonable request.
